# A novel differential diagnostic model for multiple primary lung cancer: Differentially-expressed gene analysis of multiple primary lung cancer and intrapulmonary metastasis

**DOI:** 10.3892/ol.2015.2880

**Published:** 2015-01-15

**Authors:** DALI CHEN, LONGYONG MEI, YUBIN ZHOU, CHENG SHEN, HUAN XU, ZHONGXI NIU, GUOWEI CHE

**Affiliations:** 1 Department of Thoracic Surgery, West China Hospital, Sichuan University, Chengdu, Sichuan, P.R. China; 2 Department of Thoracic Surgery, Daping Hospital, The Third Military Medical University, Chongqing, P.R. China; 3 Department of Pathology, West China Hospital, Sichuan University, Chengdu, Sichuan, P.R. China

**Keywords:** differentially-expressed gene mathematical model, multiple primary lung cancer, intrapulmonary metastasis

## Abstract

The incidence of synchronous multiple primary lung cancer (MPLC) is increasing. However, present diagnostic methods are unable to satisfy the individualized treatment requirements of patients with MPLC. The present study aimed to establish a quantitative mathematical model and analyze its diagnostic value for distinguishing between MPLC and cases of the histologically similar disease, intrapulmonary metastasis (IPM). The sum value of the differential expression ratios of four proteins, namely p53, p16, p27 and c-erbB2, was evaluated by immunohistochemically-staining specimens of primary cancers, second separate cancers, metastatic lymph nodes and metastatic cancers. The sum value of the differential expression ratio of the four proteins from the primary tumor and the lymph-node metastasis or metastatic cancer was <90 in the 11 patients with a single metastatic cancer and in the 30 patients with lymph-node metastasis, but was >90 in the 14 patients with different histological types of MPLC. Therefore, a quantitative differentially-expressed gene mathematical model was established as follows: Sum of the differential expression ratios = p16T1 − T + p27T1 − T2 + C-erbB2T1 − T2 + p53T1 − T2, where T1 is the primary cancer and T2 is the lymph node metastasis, metastatic cancer or the second separate cancer. The quantitative differentially-expressed gene mathematical model is considered to be a useful tool for distinguishing between MPLC and IPM.

## Introduction

The rate of morbidity resulting from multiple primary lung cancer (MPLC) is increasing, and in order to devise an effective therapeutic strategy, it is important to distinguish between MPLC and intrapulmonary metastasis (IPM). The incidence of synchronous MPLCs in a reported clinical series was between 1 and 7% (1). Another study reported that up to 10% of patients who survive a primary lung carcinoma will go on to develop a second primary lung tumor (2). A consensus exists regarding the therapeutic schedule of MPLC, which suggests that surgical treatment confers improved patient prognoses compared with chemotherapy. Therefore, a correct differential diagnosis of MPLC, as opposed to IPM, is conducive to effective individualized treatment. However, based solely on the Martini and Melamed criteria (3), which is widely used in clinical settings, it can be challenging to correctly diagnose MPLC. Furthermore, several cancers, including lung cancers, arise as a result of an accumulation of different genetic and epigenetic alterations (4,5). In a number of organs, carcinogenesis is considered to be a multistep process due to the accumulation of several sequential molecular abnormalities. A previous study identified that the overall frequency of the loss of heterozygosity (LOH) within cell clones progressively increased as the severity of histopathological changes progressed from hyperplasia to dysplasia to carcinoma *in situ* (6). Furthermore, the incidence of LOH has been demonstrated to increase along with the histological progression of lung adenocarcinoma (7). The definition of field cancerization has been extended to include cases of multiple primary tumors of the entire upper aerodigestive tract (8). The pathogenesis of multiple primary tumors and metastatic tumors is fundamentally different. Tissues in different fields may develop a unique genetic phenotype under the action of the same carcinogen (e.g., cigarettes) and proceed to form multiple primary tumors. By contrast, metastatic tumors are formed via the hematogenous and/or lymphatic metastasis of primary tumors. Primary and metastatic tumors exhibit a similar origin of clonality. The identification of molecular and genetic variations between tumors will allow for the differential diagnosis of MPLC and IPM. Recent advances in the study of molecular tumorigenesis have demonstrated that the genetic alterations acquired during tumor progression may act as potentially useful markers during clonality analysis. Certain studies have suggested that the gene mutational analysis of tumors could be a supplementary method to distinguish between MPLC and IPM (9–11). We have formulated two inclusion criteria in order to identify optimal genetic markers for use during clonality analysis: i) A commonly occurring and independent mutation that occurs in the early stages of disease and is maintained throughout tumor progression; and/or ii) a prognostic marker that is able to evaluate tumor progression. In total, four genetic markers, p53, p16, p27 and c-erbB2, were selected in order to examine the differences in clonality between two separate tumors from the same patient by immunohistochemical (IHC) staining. In addition, the study aimed to establish a quantitative differentially-expressed gene mathematical model to discriminate between cases of MPLC and IPM.

## Materials and methods

### Patients and clinical features

Of the 111 consecutive patients with primary lung cancer who had undergone a surgical resection between August 1999 and December 2009 at the Department of Thoracic Surgery, West China Hospital, Sichuan University (Chengdu, China), 50 patients were diagnosed with MPLCs according to the Martini and Melamed criteria (3). Of these patients, 36 exhibited MPLCs of the same histological type, including 34 patients with synchronous MPLCs and two with metachronous MPLCs, while 14 presented with MPLC of a different histological type. Finally, the 36 patients with MPLCs of the same histological type, in which paraffin sections of all tumors were available, were enrolled in the present study. In addition, 20 patients diagnosed with IPM during the same period, according to the Martini and Melamed criteria, were included. In total, 30 patients with lymph node metastasis, 11 with distant metastasis (eight brain metastases, two bone metastases and one adrenal metastasis) and 14 MPLC patients with different histological types were selected as negative or positive controls for the expression analysis of the four proteins between primary tumors and metastases. The clinicopathological data were obtained from a retrospective chart review. The tumor stage was classified according to the 2009 revision of the International System for Staging Lung Cancer (12). The characteristics of the patients with MPLCs, IPM or lymph node metastasis are shown in Table I. The experiments were approved by the West China Hospital Ethics Committee (no. 201333) and all participating patients provided informed consent.

### IHC staining

Four proteins, p53, p16, p27 and c-erbB2, which have been demonstrated to be independent prognostic factors for non-small cell lung cancer (NSCLC) (13–16), were selected for the differential diagnostic analysis of MPLC and IPM. IHC staining was performed using serial sections obtained from the same paraffin-embedded blocks. The specimens were stained with hematoxylin and eosin in order to confirm the histological diagnosis. IHC staining was performed using the streptavidin-biotin-peroxidase complex method. For the antigen retrieval, sections were briefly immersed in a citrate buffer (0.01 mol/l citric acid; pH 6.0) and then incubated for 25-min intervals at 100°C in a microwave oven. Next, the sections were incubated with a monoclonal mouse anti-p53 antibody (dilution, 1:100; sc-6243, Santa Cruz Biotechnology, Dallas, TX, USA), a polyclonal rabbit anti-p16 antibody (dilution, 1:200; ab54210, Abcam, Cambridge, MA, USA), a monoclonal mouse anti-p27 antibody (dilution, 1:250; ab32034 Abcam) and a monoclonal mouse anti-c-erbB2 antibody (dilution, 1:100; ab2428, Abcam) overnight in a cold room using a labeled streptavidin biotin kit (Dako LSAB kit; Dako, Carpinteria, CA, USA). The antibodies were diluted in phosphate-buffered saline containing 2% bovine serum albumin.

### Evaluation of the stained specimens

Appropriate positive and negative controls were selected for use in the present study. The slides were independently analyzed by two of the authors who were blinded to the clinicopathological data. A positive result for p53, p16, and p27 expression was defined as the presence of nuclear staining, whereas a positive result for c-erbB2 expression was defined as the appearance of cell membrane staining. Subsequent to the IHC detection of p53, p16, p27 and c-erbB2 in each of the specimens, the percentage of immunoreactive tumor cells in five different randomly-selected fields (magnification, ×400) was recorded. The final value for the percentage of positive tumor cells was calculated as the average of the positively-immunostained cells. The extent of immunostaining was scored according to the percentage of positive cells in each tumor specimen as follows: No staining, 0; 1–10% staining, 10; 11–20% staining, 20; 21–30% staining, 30; 31–40% staining, 40; 41–50% staining, 50; 51–60% staining, 60; 61–70% staining, 70; 71–80% staining, 80; 81–90% staining, 90; and 91–100% staining, 100.

## Results

### Establishment of the quantitative mathematical model based upon the differentially-expressed gene analysis and its application in the diagnosis of MPLC

**Figure 1:**
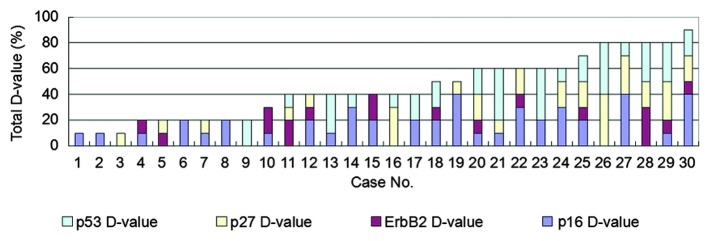
Immunohistochemistry was used to reveal the protein expression of four genes in the primary tumors and metastatic lymph nodes. The sum of the differential expression values (the D-value) between primary lung tumors and metastatic lymph nodes was <90.

**Figure 2:**
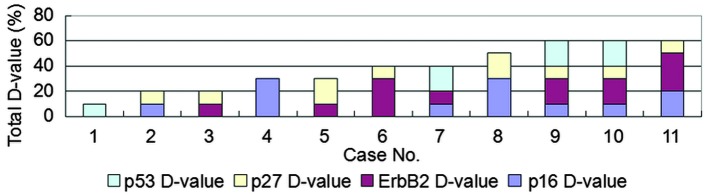
Immunohistochemistry was used to reveal the protein expression of four genes in the primary tumors and single metastatic foci. The sum of the differential expression values (the D-value) between the primary lung tumors and single metastatic foci was <90.

**Figure 3:**
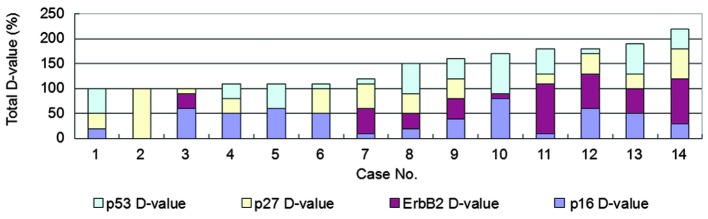
Immunohistochemistry was used to reveal the protein expression of four genes in the primary tumors and in the second tumor in cases of multiple primary lung cancers of different histological types. The sum of the differential expression values (the D-value) between the primary tumors and the second tumor in cases of multiple primary lung cancers of different histological types was >90.

First, the differential expression of the four proteins in the the primary tumors and metastatic lesions of 30 patients with lymph node metastasis and in 11 patients with distant metastasis were analyzed and subsequently served as a negative control. The differential expression of p53, p16, p27 and c-erbB2 was compared between the primary lung tumors and the metastatic tumors in the lymph nodes of the 30 patients (Table II). The sum value of the differential expression of the four proteins ranged between 10 and 90 (Fig. 1). Next, the differential expression of the four proteins was compared between the primary lung tumors and the distant metastases. The sum value of the differential expression of the four proteins ranged between 10 and 60 (Figs. 2 and 6). The maximum sum value of the differential expression ratios of the four proteins diagnosed as the same histological type of MPLC was ≤90. By contrast, the sum value of the differential expression ratios of the four proteins ranged between 100 and 220 in the 14 patients diagnosed with MPLCs of different histological types (Figs. 3 and 6). Therefore, it was hypothesized that in the case that the difference between two tumors exceeded a score of 90, the tumors were likely to be different, i.e., MPLCs.

On the basis of the experimental data, a quantitative differentially-expressed gene mathematical model was established as follows: Sum of the differential expression ratios = p16T1 − T2 + p27T1 − T2 + C-erbB2T1 − T2 + p53T1 − T2, where T1 is the primary cancer and T2 is the lymph node metastasis, metastatic cancers or the second separate cancers. According to the experimental results, tumors can be re-diagnosed as metastatic when the sum of the differential expression ratios of the four proteins does not exceed the reference value of 90, and as MPLCs when the value does exceed 90 (Table III).

### Results of de novo diagnosis based upon a differentially-expressed gene analysis mathematical model in MPLCs of the same histological type

**Figure 4:**
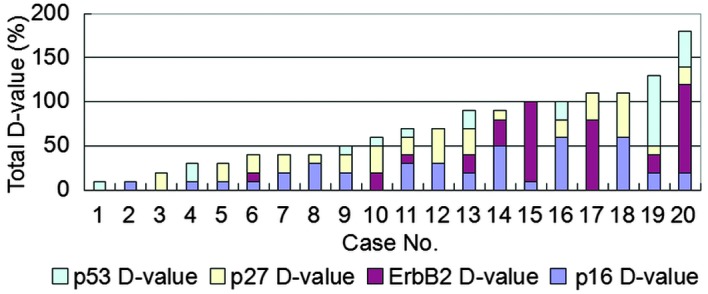
Immunohistochemistry was used to reveal the protein expression of four genes in the primary tumors and in the second tumor in cases of multiple primary lung cancer (MPLC) with the same histological type. According to the new classification, case nos. 1 to 14 were newly-classified as intrapulmonary metastases and case nos. 15 to 20 were newly-classified as MPLCs. The sum of the differential expression values (the D-value) between the primary tumors and the second tumor in cases of MPLC with the same histological type was <90 for cases 1–14 and >90 for cases 15–20.

Of the 36 patients with the same histological type of MPLC, who were clinically diagnosed according to the Martini and Melamed criteria (3), the sum value of the differential expression ratio was >90 in 29 patients (80.5%), and <90 in seven patients (19.5%) (Figs. 4 and 6). According to the model, 29 of the 36 patients (82.0%) were diagnosed *de novo* with newly-classified MPLCs and seven with newly-classified IPM.

### Results of de novo diagnosis based upon a differentially-expressed gene analysis mathematical model in lung cancers with IPM

**Figure 5:**
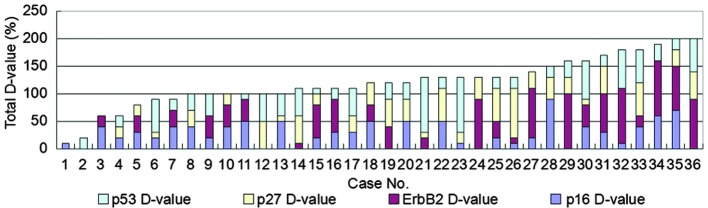
Immunohistochemistry was used to reveal the protein expression of four genes in the primary tumors and in the second tumor in lung cancers with intrapulmonary metastasis. According to the new classification, case nos. 1 to 7 were newly-classified as intrapulmonary metastasis and case nos. 8 to 36 were newly-classified as multiple primary lung cancers.

**Figure 6:**
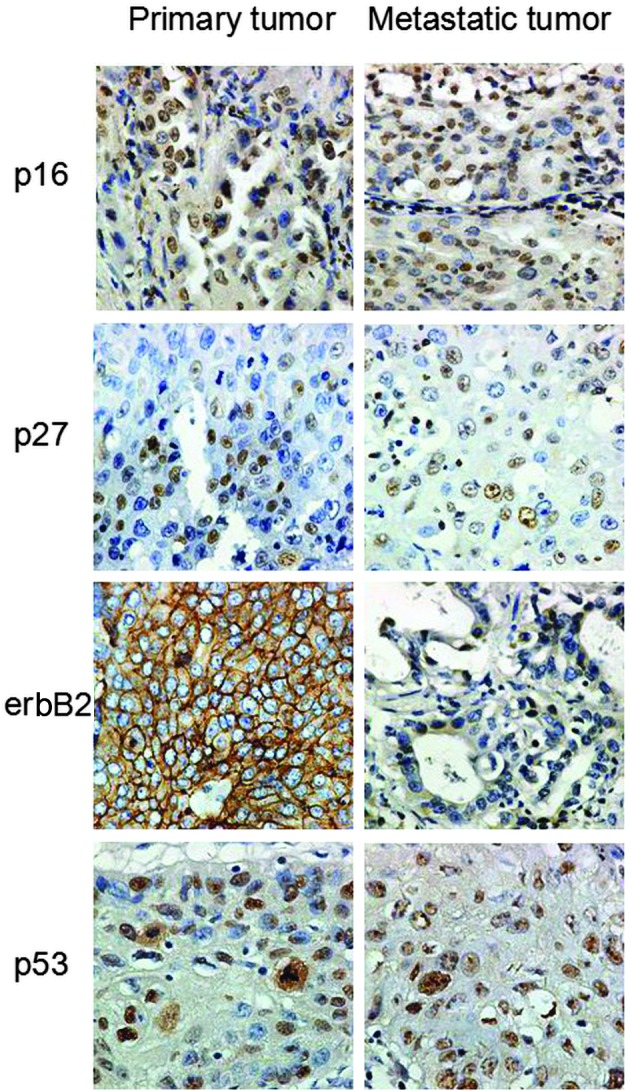
Immunostaining revealing the expression status of p16, p27, C-erbB2 and p53 in primary and metastatic tumors of non-small cell lung cancer. Positive expression revealed by brown-yellow nuclear staining (magnification, ×175).

Of the 20 patients with IPM who were clinically diagnosed according to the Martini and Melamed criteria (3), 14 (70.0%) had a sum value of ≤90 for the expression ratios of the four proteins. According to the same criterion, 14 of the 20 patients were diagnosed *de novo* with newly-classified IPM, and six with newly-classified MPLCs (Figs. 5 and 6). In total, three of the six patients (50%) with IPM demonstrated unilateral lung lobe lesions, and the other three patients presented with bilateral lung lobe lesions. The pathological stage was diagnosed *de novo* as being between T4 and T1 among three of the six patients, and as M1 to T2 in the rest.

### Differences in the diagnostic consistency of the mathematical model, based on differentially-expressed gene analysis and clinical diagnosis

In total, 29 of the 36 MPLC patients were diagnosed with newly-classified MPLC and the remaining seven with newly-classified IPM. Furthermore, 14 of the 20 cases of IPM were diagnosed with newly-classified IPM and the other six with MPLC. Overall, 35 patients with multifocal lung cancer were diagnosed *de novo* with newly-classified MPLC, and 21 with newly-classified IPM (Table III).

## Discussion

At present, individuals with lung cancer have an increased risk of developing a second lung tumor. Cases of MPLC are distinguished by the presence of a secondary neoplasm. It may be easy to diagnose cases of MPLC that exhibit different histological types. Multiple, anatomically distinct, but histologically similar lung cancers are commonly identified in the same patient. Often, it can be challenging to distinguish between cases of MPLC and IPM. The diagnostic criteria for MPLC was proposed by Martini and Melamed (3) and states that: i) MPLC tumors must occur in separate lobes or in different regions of the same lobe, ii) neoplasms may originate from different types of carcinomas *in situ* and demonstrate distinct histological types, and iii) no metastasis should be evident in the lymphatic system or in any other organs. However, not all patients can be classified in accordance with these guidelines. Patients with clinically diagnosed MPLCs occasionally demonstrate extremely poor five-year survival rates (0–44%), even at stage I of the disease (3,17–19). This variation in prognosis is believed to be the result of the different biological behaviors of the tumors. These results suggest that a number of patients with clinically diagnosed MPLCs may possess metastatic lesions. This indicates a potential limitation in the Martini and Melamed criteria (3), which, at present, is widely used for the clinical diagnosis of MPLCs.

A universal agreement regarding the particular approach that should be adhered to for the diagnosis of MPLC is yet to be established. Therefore, biological analyses are considered to be a useful approach for distinguishing between cases of MPLC and IPM, and for determining the correct biological stage of the lung cancer. In order to overcome this limitation, the use of clonal analyses for different tumors has been reported to discriminate between MPLCs and IPM. Previous studies have demonstrated that multiple gene analyses are able to identify the clonality in a combination of multiple gene mutations, including a p53 gene mutation, a K-ras mutation and/or LOH (11,20–28). In order to differentiate between multifocal tumors and IPM, Chang *et al* (29) evaluated p53 somatic aberrations in MPLCs. Of the 58 patients included in the study, 22 (37.9%) were identified as having the same clonality and 28 (48.3%) as having different clonalities. Furthermore, it was revealed that the occurrence of lymph node metastasis was more common in lesions with the same clonality.

In the present study, IHC staining was performed in order to distinguish between MPLCs and IPM. The IHC expression levels of p53, p16, p27 and c-erbB2 were revealed to be significant prognostic factors for cases of lung cancer. The transcription factor, p53, is activated in response to DNA damage and is involved in cell cycle regulation, the induction of apoptosis and DNA repair. However, mutated forms of p53 are unable to effectively retain these particular functions. A mutated version or an overexpression of the p53 gene is an unfavorable prognostic factor that is observed in ~50% of patients with NSCLC (30). In a previous study, the presence of somatic mutations or an overexpression of p53 were identified in ~23% and ~65% of patients with NSCLC, respectively. Furthermore, p53 has been extensively investigated as a prognostic marker in cases of NSCLC, and the majority of results indicate that alterations in p53 are associated with a poor prognosis (31). The reproduction of human lung adenocarcinoma phenotypes in the flanks of nude mice has been successfully completed by introducing a p53 gene alternation (32). The p16 gene is also a tumor suppressor gene, which negatively regulates cell cycle progression by inhibiting cyclin-dependent kinases (CDK) 4/6. Homozygous deletions (HDs) of p16 have been frequently detected in lung cancer patients. In a previous study, HDs were detected in eight of 28 (28.6%) primary tumor patients, including two of eight (25.0%) non-invasive bronchioloalveolar carcinomas, and five of 22 (22.7%) brain metastases (33). In another study, abnormal hypermethylation of the p16 promoter was detected in several tumors types, and was revealed to be inactivated in 40–70% of patients with NSCLC (34). The contribution of p16 deregulation via alterations in methylation during the carcinogenic process has been extensively investigated. p16 hypermethylation is considered to be an independent prognostic factor for poor patient outcomes (35). p27 is a CDK inhibitor, which is involved in the regulation of the cell cycle. By inhibiting retinoblastoma phosphorylation, p27 is able to suppress the progression of the cell cycle from the G_1_ phase to the S phase. A reduced expression of p27 is observed in 70–80% of patients with NSCLC. In previous studies, this particular reduced-expression group demonstrated a poorer prognosis compared with patients from a positive-expression group (15,36,37). By contrast, high p27 expression is associated with an improved prognosis (38). The c-erbB-2 (HER-2/neu) proto-oncogene codes for transmembrane receptor tyrosine kinases, such as epidermal growth factor and human epidermal growth factor receptor 2, 3 and 4, which are members of the class 1 receptor tyrosine kinase family. An overexpression of c-erbB2 is often observed in patients with NSCLC. In a previous study, c-erbB2 overexpression was identified in 37% of lung adenocarcinomas cases that were associated with a higher disease stage and a positive nodal status. Therefore, c-erbB2 has been suggested to be a potential tumor progression marker in NSCLC patients, and one that can be observed at the protein level (39,40).

Two important mechanisms have been proposed, through which histologically-similar, multifocal tumors are believed to arise: i) A single clonal event occurs, which results in a tumor that subsequently spreads within one or two lungs; and ii) multiple tumors arise independently in a carcinogen-damaged field (41). The difference in protein expression between the histologically-similar tumors was hypothesized to be larger in MPLC patients, due to the various clonal origins. The D-value of protein expression in IPM patients, however, would be smaller. According to the concept of field cancerization, tissues from different fields may conduct similar or dissimilar DNA damage under the control of a carcinogen. The possibility that separate MPLC tumors may contain a similar genotype could be a default from the probability theory. When two or more separate tumors share one genotype, they are likely to be IPMs (41,42).

Based on our preliminary experiments and the results of a literature review, the reliability of a single gene marker appeared to be low. Therefore, four markers, p53, p16, p27 and c-erbB2, were selected in order to distinguish between cases of MPLC and IPM, according to the early or late emergence of the gene mutation, the stability of the mutation and the correlation with prognosis. In the present study, the results indicated that when the difference between two tumors was >90, the patient could be newly classified as having MPLC. The 14 patients diagnosed with MPLCs of different histological types had a sum value of >90 for the differential expression ratios of the four proteins, which was concomitant with our hypothesis. By contrast, when the difference was <90, the patient was newly classified with IPM. Therefore, 36 of the MPLCs cases of the same histological type and 20 of IPM (based on the Martini and Melamed criteria) were reclassified using this novel criteria.

The results of the present study demonstrated that IHC analyses of differential protein expression profiles of multiple genes can be used to indicate the clonal origins of multiple separate tumors, and therefore facilitate the discrimination between a secondary primary cancer and IPM. As a classical pathological examination method, IHC has a number of merits, including convenience and sensitivity. For the patients diagnosed with MPLCs, particularly those with the same histological type, it was challenging to determine a correct diagnosis of MPLC or IPM based entirely on the Martini and Melamed criteria (3). Therefore, an IHC test should be performed in order to confirm the correctly diagnosed ratios. The quantitative differentially-expressed gene mathematical model is considered to be a useful approach for distinguishing between MPLCs of the same histological type and IPM. The precise discrimination between MPLC and IPM should enable rationalized treatment strategies, and improve the prognoses of the affected patients. However, as the number of analyzed cases in the present study was relatively small, future studies with larger cohorts will be required in order to confirm these results.

## Figures and Tables

**Table I:** Tumor characteristics.

A, Intrapulmonary, distant and lymph node metastases			

Characteristics	Intrapulmonary metastasis	Distant metastasis	Lymph node metastasis
No. of patients	20	11	30
Age, years (range)	62 (46–74)	55 (42–70)	60 (38–72)
Gender, n (%)			
Male	12 (60)	6 (54.5)	22 (73.3)
Female	8 (40)	5 (45.5)	8 (26.7)
Second cancer, n (%)			
Metachronous	3 (15)		
Synchronous	17 (85)		
No. of tumors			
2	20		
3			
Histological type, n			
Adenocarcinoma	15	4	17
Squamous cell carcinoma	5	4	10
Other		3	3
p stage (2009 UICC)^b^, n			
IA			
IB			
IIA			6
IIB			4
IIIA	10		18
IIB	7		1
IV	3	11	1

B, MPLC			

Characteristics	MPLC total	Same histological type	Different histological type
No. of patients	50	36	14
Age, years (range)	61 (38–80)		
Gender, n (%)			
Male	34 (68)		
Female	16 (32)		
Second cancer, n (%)			
Metachronous	28 (56)		
Synchronous	22 (44)		
No. of tumors			
2	34	13	
3	2^a^	1	
Histological type			
Adenocarcinoma		33	11
Squamous cell carcinoma		3	11
Other			3
p stage (2009 UICC)^b^			
IA		15	2
IB		12	2
IIA		2	3
IIB		6	1
IIIA		1	2
IIIB			4
IV			

a The superior and inferior lobes of the left lung contained an adenocarcinoma, with a previous history of gastric adenocarcinoma. The other two masses in the middle and inferior lobe of right lung were identified during a pre-operative examination for cervical cancer.

b According to the tumor-node-metastasis classification.

MPLC, multiple primary lung cancer; p stage, pathological stage; UICC, Union for International Cancer Control.

**Table II:** Immunohistochemical protein expression of the four genes in primary tumors and metastatic lymph nodes.

Case no.	p16 D-value	ErbB2 D-value	p27 D-value	p53 D-value	Total D-value
1	10	10	0	0	20
2	10	10	20	20	60
3	0	20	10	10	40
4	20	10	10	0	40
5	40	10	20	20	90
6	10	0	0	30	40
7	30	0	0	10	40
8	0	0	40	40	80
9	40	0	10	0	50
10	10	0	0	0	10
11	10	0	10	40	60
12	0	10	10	0	20
13	20	10	0	20	30
14	30	10	20	0	60
15	40	0	30	10	80
16	20	0	0	40	60
17	10	0	0	0	10
18	20	0	0	0	20
19	20	10	20	20	70
20	0	0	10	0	10
21	20	20	0	0	40
22	0	0	30	10	40
23	10	20	0	0	30
24	10	0	10	0	20
25	0	30	20	30	80
26	20	0	0	20	40
27	30	0	20	10	60
28	10	10	30	30	80
29	20	0	0	0	20
30	0	0	0	20	20

D-value, the sum of the differential expression ratios.

**Table III:** Difference between the Martini and Melamed criteria and the mathematical model, which is based upon differentially-expressed gene analysis, in MPLC.

	Clinical diagnosis (Martini and Melamed criteria)		
	
Differentially-expressed gene analysis	MPLC	IPM	Total
MPLC	29	6	35
IPM	7	14	21
Total	36	20	56

Martini and Melamed criteria (3); MPLC, multiple primary lung cancer; IPM, intrapulmonary metastasis.
